# Effect of preoperative patient education and simulated mouth breathing training on opioid requirements in the post-anesthesia care unit after nasal surgery: a randomized controlled study

**DOI:** 10.1186/s12871-023-02310-x

**Published:** 2023-10-20

**Authors:** Yu Jeong Bang, Sojin Kim, Jin Kyoung Kim, Hara Kim, Seungmo Kim, Chi Song Chung, Seung Yeon Yoo, Heejoon Jeong, Boram Park, Sang Hyun Lee

**Affiliations:** 1 Department of Anesthesiology and Pain Medicine, Samsung Medical Center, Sungkyunkwan University School of Medicine, 81 Irwon-ro, Gangnam-gu, Seoul, 06351 Korea; 2 Biomedical Statistics Center, Research Institute for Future Medicine, Samsung Medical Center, Seoul, Korea

**Keywords:** Nasal surgical procedures, Pain, Postoperative, Analgesics, opioid, Opioid sparing, Preoperative simulated training, Patient education

## Abstract

**Background:**

A simulated education, prior to surgery about postoperative nasal stuffiness and ease of breathing through the mouth may help patients tolerate discomfort after nasal surgery. This study aimed to investigate the effect of preoperative simulated education on immediate postoperative opioid requirements in patients undergoing elective nasal surgery.

**Methods:**

This randomized controlled trial of 110 patients undergoing nasal surgery randomly allocated patients into either a control (group C) or an education group (group E). One day before surgery, patients in group E were intensively trained to breathe through the mouth by using a nasal clip, with informative explanations about inevitable nasal obstruction and discomfort following surgery. Patients in group C were provided with routine preoperative information. Total intravenous anesthesia (TIVA) with propofol and remifentanil was used for anesthesia. No further opioid was used for analgesia intraoperatively. The primary outcome was index opioid (fentanyl) requirements at the post-anesthesia recovery unit (PACU). Secondary outcomes were emergence agitation, pain scores at the PACU, and postoperative recovery using the Quality of Recovery-15 (QoR15-K).

**Results:**

The rate of opioid use in the PACU was 51.0% in the group E and 39.6% in the group C (p = 0.242). Additional request for analgesics other than index opioid was not different between the groups. Emergence agitation, postoperative pain severity, and QoR15-K scores were comparable between the groups.

**Conclusion:**

Preoperative education with simulated mouth breathing in patients undergoing nasal surgery did not reduce opioid requirements.

**Trial registration:**

KCT0006264; 16/09/2021; Clinical Research Information Services (https://cris.nih.go.kr).

**Supplementary Information:**

The online version contains supplementary material available at 10.1186/s12871-023-02310-x.

## Introduction

Nasal surgery is a commonly performed procedure, with more than 60,000 cases annually in Korea [1]. Yet standard postoperative analgesic care after nasal surgery has not been established in clinical practice [1].

Patients receiving nasal surgery often have symptoms of long-standing chronic nasal fullness, headache which is distinguished from that of neurogenic origin, breathing through the mouth, and depressive mood or anxiety [2, 3]. Expectations of these patients may be nasal clearness or ease of breathing after surgery, but in the immediate postoperative period, many experience even more nasal fullness with headache and discomfort due to nasal packing or mucosa edema, and request for analgesics [2, 4]. Clinicians often prescribe opioids to relieve these symptoms [5], although postoperative pain in nasal surgery is reported to be generally tolerable with non-steroidal anti-inflammatory drugs (NSAIDs) or acetaminophen [6, 7].

Opioids are potent analgesics but should be carefully prescribed for intense perioperative pain because of their undesirable and potentially lethal side effects such as postoperative nausea and vomiting, constipation, respiratory depression, and addiction to opioids [8]. For these reasons, relentless efforts are put into reducing the use of opioids and promoting multimodal analgesia using non-opioids and/or regional nerve block in perioperative analgesic care. Moreover, non-pharmacological methods of preoperative patient education are investigated to reduce opioid use [9, 10]. In one study with colorectal surgeries requiring urethral catheterization (with tetracaine mucilage), the incidence of catheter-related bladder discomfort and postoperative pain during the first postoperative 6 h was significantly lower in patients who received illustration education before surgery compared to those who did not [9]. Another study on the preoperative education of the effects of endogenous beta-endorphin significantly reduced postoperative opioid consumption in breast augmentation surgery [10].

We hypothesized that preoperative education with simulation training using a nasal clip might improve patients’ understanding and awareness of the postoperative uncomfortable nasal stuffiness, and help them better tolerate the condition and reduce requests for opioids. Therefore, in this randomized, controlled study, we aimed to determine whether preoperative education with simulated training could reduce opioid consumption or discomfort in patients undergoing nasal surgery. We also investigated the presence of emergence agitation, the severity of pain, and patients’ satisfaction with their recovery before the nasal packing was removed.

## Materials and methods

### Ethics

This randomized controlled study was performed at Samsung Medical Center in Seoul, Korea, between 22/06/2021 and 9/11/2021. The Samsung Medical Center Institutional Review Board approved this study (SMC 2021-05-098-001) on 1/06/2021. The study was registered with Clinical Research Information Services (https://cris.nih.go.kr, CRIS identifier: KCT0006264) on 16/06/2021. The study was conducted in accordance with the principles of the Declaration of Helsinki and the International Conference on Harmonization of Good Clinical Practice guidelines. Written informed consent was obtained from each participant prior to participation.

### Study population

We enrolled adult patients aged 18–75 years old, of American Society of Anesthesiologists (ASA) physical status classification I to II, and who were scheduled for elective nasal surgery. The included surgeries were endoscopic sinus surgery, septoplasty, and turbinoplasty. The exclusion criteria were ASA physical status of III or above, inability to communicate in Korean, and rhinoplasty.

### Randomization and blinding

The enrolled patients were allocated into either the control group (group C) or the education group (group E) at a ratio of 1:1. A randomization sheet with a block size of four was generated using a web service (www.randomizer). The allocation information was sealed in opaque envelopes numbered with the randomization sequence and stacked in the operating room. An investigator (YJB) opened each envelope in sequence and then visited the participants and provided instruction and education.

An independent anesthesiologist who was not involved in patient education or mouth-breathing training was responsible for inducing and supervising the entire anesthesia process. Two investigators (SHL, SK) observed patient emergence and evaluated patient consciousness using the Richmond Agitation-Sedation Scale (RASS) before and immediately after extubation and recorded the time to awakening, time to extubation, and the presence and extent of any adverse events during emergence from anesthesia. During the PACU stay, an independent attending physician evaluated patient consciousness and managed their physical status according to predetermined protocols. One blinded researcher (CSC) examined patient discomfort with oral breathing after surgery, pain severity, and satisfaction with recovery before the nasal packing was removed on the first postoperative day.

### Intervention

**Fig. 1:**
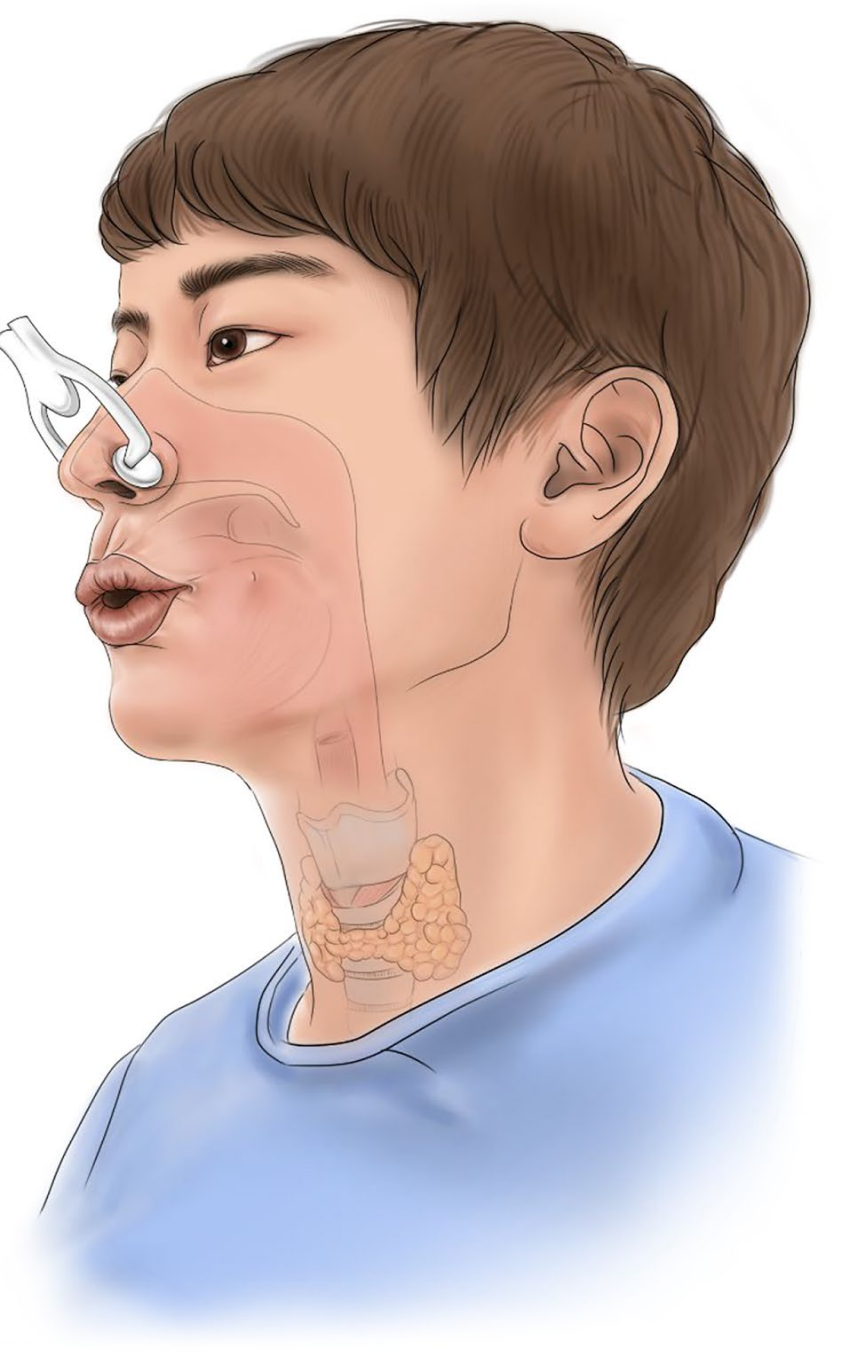
Schematic drawing of preoperative simulated education and training with nasal clip

All patients were informed about the standard general anesthesia and perioperative management process. The participants in the education group (group E) received simulated mouth-breathing training with a nasal clip. The training involved a 30-minute session in which an experienced board-certified anesthesiologist (YJB) provided an in-depth education about mouth breathing. The decision of a 30-minute session was based on the previous studies [10, 11] and the practical consideration of a simple training process. First, patients were told that nasal breathing is impractical immediately after surgery, and therefore mouth breathing is mandatory when awakening from anesthesia. Patients were encouraged to practice mouth breathing with a disposable nose clip at least three times before the surgery, once every hour, to gain awareness about their postoperative situation in advance (Fig. 1). On the day of the surgery, the patients practiced mouth breathing for the last time under the guidance of the researcher just before entering the operating room.

### Anesthesia and perioperative management

After applying full monitoring (EKG, NIBP, SpO_2_) according to the standard anesthesia protocol, the attending anesthesiologist induced anesthesia, including endotracheal intubation and intravenous line placement, if applicable. A target-controlled infusion (Orchestra^®^ with Base Primea, Fresenius Kabi) of propofol and remifentanil was used for induction and maintenance of anesthesia to keep the anesthetic depth on the bispectral index to 35–50. There was no further use of other analgesics during surgery. The neuromuscular blockade was maintained to ≤ two counts of train-of-four during surgery. Nasal packings were performed at the surgical sites of the nasal cavity, but sometimes bilateral nasal packings were inserted in unilateral ESS or septo-turbinoplasty at the discretion of the surgeon. At the end of the surgery, the neuromuscular blockade was reversed with 0.25 mg/kg of pyridostigmine or sugammadex at the discretion of the anesthesiologist.

After emergence from anesthesia and extubation, the patients were transferred to the PACU, where they stayed until they reached a modified Aldrete’s score ≥ 9.

The postoperative analgesic strategy was that if the patients reported pain of ≥ 4 on a numeric rating scale (NRS), 0.5 mcg/kg of fentanyl was given according to a pre-determined protocol. In the surgical ward, an independent otolaryngologist managed each patient according to our institutional protocol.

### Outcomes

Patient characteristics (age, sex, height, weight, ASA class, comorbidities, smoking history, and previous operative history) were collected. The primary outcome was fentanyl (0.5 ug/kg) requirements in the PACU. For the secondary outcome, an additional request for analgesics other than the index opioid was counted (other opioids, acetaminophen or NSAID).

Other intraoperative covariates for secondary outcomes were as follows: (1) the level of consciousness upon awakening, as assessed by the RASS immediately before and after extubation [12]; (2) time to emergence (from the discontinuation of the anesthetic to awakening) and arousal, as assessed by a score of ≥ 3 on the Observer’s Assessment of Alertness/Sedation Scale [13]; (3) time to extubation (from the discontinuation of the anesthetic to tracheal extubation); and (4) severity of cough (cough before extubation upon awakening is evaluated using four grades [14]: 0, no cough; 1, single cough; 2, more than one episode of un-sustained coughing, 3, sustained and repetitive coughing with head lift). Total anesthesia duration, surgery duration, medications used during anesthesia, and vital signs were collected through medical records. PACU variables were also collected: postoperative RASS, postoperative nausea and vomiting (PONV), and postoperative pain as rated on an NRS.

Our participants were asked to fill out surveys about recovery, discomfort, and satisfaction on the postoperative day 1. The Quality of Recovery-15 questionnaire (QoR15) is a validated questionnaire specifically designed to evaluate the health status during the early postoperative period, with higher scores indicating a better recovery [15]. We used the Korean version of the Quality of Recovery-15 questionnaire (QoR15-K) to assess postoperative recovery [16]. The NRS of 0 to 10 was also queried for patients’ satisfaction and discomfort with nasal packing after surgery. The Nasal Obstruction Symptom Evaluation score (NOSE) scale is a validated tool for evaluating nasal obstruction-related discomfort both before and after nasal surgery [17–19], and it was used to rate discomforts of nasal obstruction [19].

### Sample size calculation and statistical analysis

The sample size was calculated on the basis of our institutional data. In the review of medical records of patients who underwent nasal surgery from 13 to 2021 to 13 May 2021 at our institution, 55.3% of patients received opioids at the PACU. Assuming that the opioid requirement was reduced by 50% in the educated group, 50 patients in each group were necessary to achieve a power threshold of 80% at the significance level of 5% (two-tailed test). With an expected dropout rate of 10%, 110 patients (55 patients per group) were required.

Categorical variables are presented as frequencies and percentages (%), and were analyzed by Chi-square test or Fisher’s exact test as appropriate. Continuous variables were compared by a two-sample t-test or Wilcoxon’s rank-sum test as appropriate, after checking normality with the Shapiro-Wilk test, and are presented as a mean (standard deviation) or median (interquartile ranges). Intention to treat analysis was carried out to compare index opioid (fentanyl) requirements between the groups in the PACU. Risk factors for the opioid requirement at the PACU were analyzed by univariable and multivariable logistic regression with backward stepwise model selection using the Akaike Information Criterion (AIC). The likelihood Ratio test and Hosmer-Lemeshow test were performed for the goodness-to-fit test. The risk was expressed as an adjusted odds ratio (aOR) and 95% confidence interval (95% CI). Statistical significance was assumed when *P*-value < 0.05. Statistics were carried out using SPSS 28.0 or SAS software, version 9.4 (SAS Institute Inc., Cary, NC, USA).

## Results

**Fig. 2:**
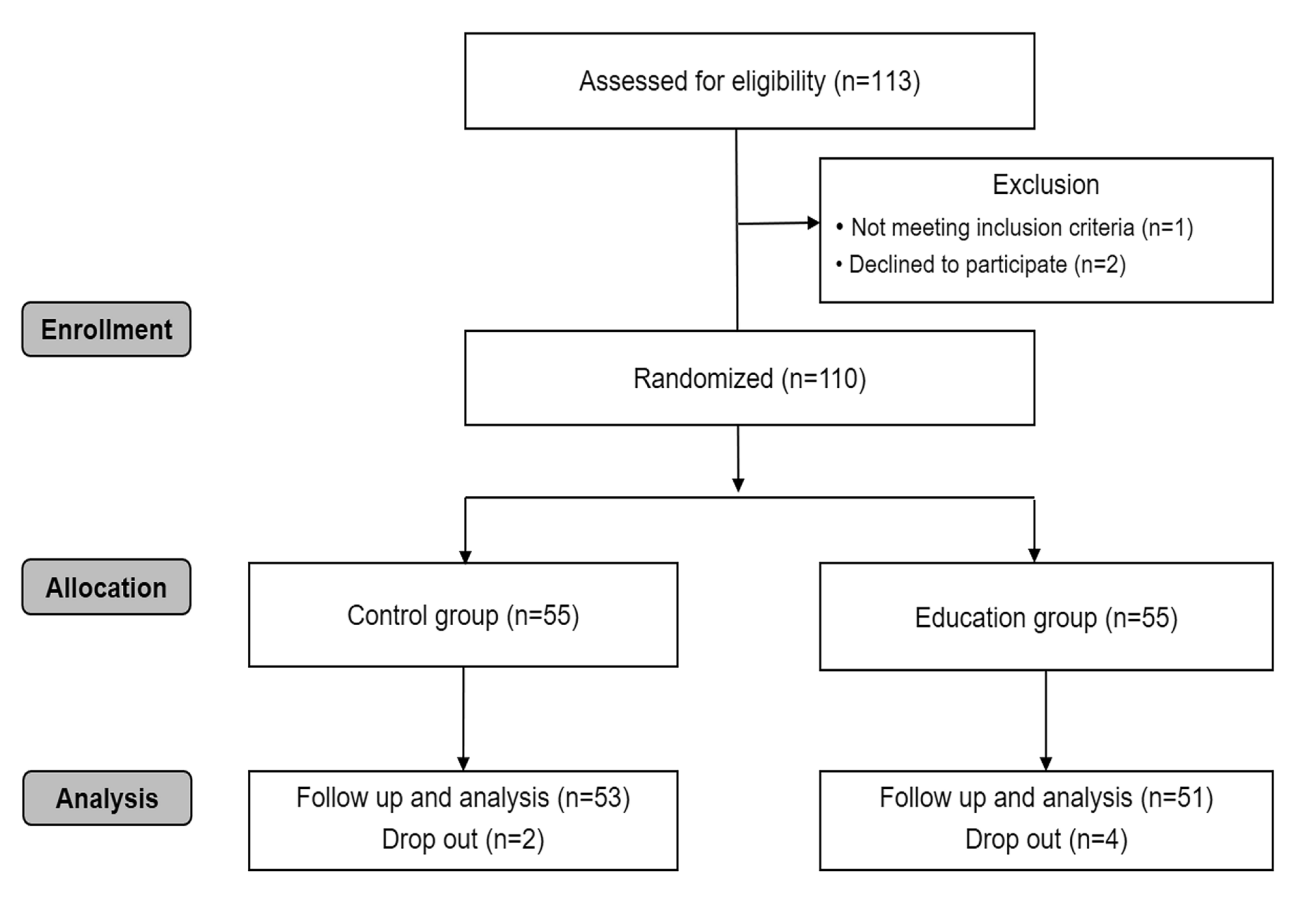
Consolidated Standards of Reporting Trials flow diagram of patients included in the study

From June 2021 to November 2021, 113 patients who underwent elective nasal surgery were assessed for eligibility (Fig. 2). Among them, two patients declined to participate in the study, and one patient did not meet the inclusion criteria. All enrolled participants (*n* = 110) were randomly allocated to the control or education group. Six participants (control group, *n* = 2; education group, *n* = 4) were considered dropouts after randomization by the predetermined criteria. The two cases in the control group were due to surgical plan alteration. In the education group, one dropout was due to the patient’s rejection of training for mouth breathing, and three were due to an unplanned change in surgical approach. Thus, 104 patients (control group, *n* = 53; education group, *n* = 51) were included in the analysis. One patient in the control group received hydromorphone (0.8 mg) intraoperatively at the end of surgery, which was a violation of protocol. We performed the final analysis according to an intention to treat analysis.

Patient demographic data and intraoperative and at emergence variables were comparable between the groups (Tables 1 and 2).

**Table 1:** Patient characteristics

Parameter	Control group (*N* = 53)	Education group (*N* = 51)	*p* value
Age (yr)	49 [39–58]	47 [ 32–57]	0.240
Height (cm)	165.6 ± 9.7	169.3 ± 9.0	0.047
Weight (kg)	67.7 ± 13.9	71.2 ± 14.3	0.204
BMI (kg·m^− 2^)	24.5 ± 3.6	24.7 ± 3.5	0.829
ASA physical status (I : II) (%)	16 : 37 (30.2% : 69.8%)	16 : 35 (31.4% : 68.6%)	0.896
Male: female	31 : 22 (58.5% : 41.5%)	37 : 14 (72.6% : 27.5%)	0.132
Comorbidity (%)			
Hypertension	10 (18.9%)	4 (7.8%)	> 0.99
Diabetes mellitus	3 (5.7%)	4 (7.8%)	0.713
Liver disease	3 (5.7%)	3 (5.9%)	> 0.99
COPD	2 (3.8%)	1 (2.0%)	> 0.99
Asthma	8 (15.1%)	4 (7.8%)	0.247
Allergic rhinitis	44 (83.0%)	41 (78.4%)	0.553
Obstructive sleep apnea	8 (15.1%)	9 (17.6%)	0.725
Ever smoker (Current and previous)	17 (32.1%)	26 (51.0%)	0.050
Alcohol user	23 (43.4%)	23 (45.1%)	0.861
Previous general anesthesia	28 (52.8%)	27 (52.9%)	> 0.99
Previous surgery	30 (56.6%)	29 (26.9%)	0.979
Nasal obstruction (none:uni:bi)	6 : 11 : 31 (12.5% : 22.9% : 64.6%)	4 : 16 : 29 (8.2% : 32.7% : 59.2%)	0.618

Values are the mean ± SD, median [IQR], or number of patients (%)

*ASA* American Society of Anesthesiologists, *COPD* chronic obstructive disease, *IQR* interquartile range, *SD* standard deviation

**Table 2:** Intraoperative variables

Parameter	Control group (*N* = 53)	Education group (*N* = 51)	Difference in means or medians (95% CI)	*p* value
***Intraoperative parameter***				
Anesthetic time (min)	128.3 ± 41.0	127.9 ± 44.6	0.4 (-16.3, 170)	0.966
Propofol (mg)	1000 [840–1360]	1060 [900–1600]	− 70 (-220, 80)	0.354
Remifentanil (mg)	0.55 [0.4–0.85]	0.6 [0.5–0.85]	0.0 (-1, 1)	> 0.99
Rocuronium (mg)	55 [50–65]	60 [50–70]	-5.0 (-10, 0)	0.034
Crystalloid (mL)	400 [350–550]	450 [350–560]	0 (-50, 50)	0.687
Surgical procedure				0.613
ESS only	14 (26.4%)	9 (17.7%)	N/A	
Septoplasty or turbinoplasty	12 (22.6%)	16 (31.4%)	N/A	
ESS with either Septoplasty or turbinoplasty	27 (50.9%)	27 (52.9%)	N/A	
Surgical site (uni/bi)	19 / 34 (35.9% / 64.2%)	17 / 34 (33.3% / 66.7%)	N/A	0.788
Nasal packing (uni/bi), *n* (%)^a^	13 / 40 (24.5% / 75.5%)	9 / 42 (17.6% / 82.4%)	N/A	0.390
Packing materials^b^				0.218
Nasopore	40 (75.5%)	43 (84.3%)	N/A	
Beschitin	4 (7.6%)	5 (9.8%)		
Both (Nasopore and Beschitin)	5 (9.4%)	3 (5.9%)		
Others	4 (7.6%)	0 (0%)		
Estimate blood loss (mL)	100 [30–200]	50 [20–200]	0 (-20, 50)	0.659
***Parameters at emergence***				
Coughing, *n* (%) (none/mild/moderate/severe)	16 / 5 / 6 / 25 (30.8% / 9.6% /11.5% /48.1%)	16 / 4 / 11 / 20 (31.4% / 7.8% / 21.6% / 39.2%)	N/A	0.559
Time to emergence (min)	11 [8–13]	11 [8–14]	-1 (-2, 1)	0.493
Time to extubation (min)	12 [10–15]	13 [10–16]	-1 (-2,1)	0.366
Agitated emergence	14 (26.9%)	10 (19.6%)	N/A	0.418
Adverse event^c^	0 (0%)	2 (3.8%)	N/A	0.238

Values are the mean ± SD, median [IQR], or number (%)

Agitated emergence was defined as RASS ≥ 1

*CI* confidence interval, *ESS* endoscopic sinus surgery, *IQR* interquartile range, *RASS* Richmond Agitation-Sedation Scale, *SD* standard deviation

^a^ In some patients, bilateral nasal packing is performed in unilateral ESS or septo-turbinoplasty at the discretion of the surgeon

^b^ Nasopore and Beschitin are both absorbable nasal packing materials. Others consists of minocell (*n* = 1) or guardcell (*n* = 3)

^c^ Two adverse events were nasal bleeding at emergence that required brief bleeding control by surgeons

Overall opioids requirement at PACU was 47 (45.2%) among 104 enrolled patients. Our primary outcome, the index opioid requirement (fentanyl) at the PACU, was observed in 21 out of 53 patients (39.6%) in group C and 26 out of 51 patients (51.0%) in group E (p = 0.245). After adjusting for confounding factors, opioid requirement between the groups was not different (aOR = 1.80; 95% CI 0.755, 4.274; p = 0.185) (Supplementary Table 1). Additional requests for analgesics at the PACU after the administration of the index opioid were noted in 3 out of 53 patients (5.7%) in group C and none (0%) in the education group (p = 0.129) (Table 3). Multivariable analysis showed that patients who received Beschitin® packing material were more likely to require opioids at PACU than patients who received Nasopore® (aOR = 19.49; 95% CI 2.225, 171.473; *p* = 0.007) (Supplementary Table 1). In the surgical wards, the QoR-15 K score representing postoperative recovery, satisfaction, and subjective discomfort were not different between the groups (Table 4).

**Table 3:** Opioid requirements and other variables in the PACU

Parameter	Control group (*N* = 53)	Education group (*N* = 51)	Difference in medians (95% CI)	*p* value
Index opioid administration^a^	21 (39.6%)	26 (51.0%)	0.793 (0.537, 1.171)	0.245
Additional analgesic request after index opioid administration^b^	3 (5.7%)	0 (0%)	N/A	0.129
Postoperative pain, NRS_max_ (0–10)	2 [2–4]	4 [2–5]	0 (-1, 0)	0.362
NRS severity^c^ (none / mild / moderate to severe)	8 / 25 / 20 (15.9% /47.2% /37.7%)	8 / 17/ 26 (15.7% / 33.3% / 51.0%)	N/A	0.322
Nasal dressing change event	9 (17.0%)	8 (15.7%)	N/A	0.858
PONV	5 (9.4%)	1 (2.0%)	N/A	0.206
RASS ≥ 1	2 (3.8%)	4 (7.8%)	N/A	0.374

Values are the median [IQR] or number (%)

Maximum degree of postoperative pain was recorded during PACU stay using a numeric rating scale (NRS_max_), 0 to 10

*CI* confidence interval, *IQR* interquartile range, *PONV* postoperative nausea and vomiting, *RASS* Richmond Agitation-Sedation Scale

^a^ Index opioid refers to fentanyl

^b^ Additional analgesic includes opioid, acetaminophen, non-steroidal anti-inflammatory drugs

^c^ Pain severity was defined using NRS: mild: 1 to 3, moderate: 4 to 6. Severe: 7 to 10

**Table 4:** Recovery variables after 12 postoperative hours

	Control group (*N* = 52)	Education group (*N* = 51)	Difference in medians (95% CI)	*p* value
QoR15-K score, 0–150	109 [87, 128]	108 [91, 128]	2 (-9, 11)	0.747
Moderate pain, 10–0	5 [3–8]	6 [4–8]	0 (-2, 1)	0.415
Severe pain, 10–0	9 [5–10]	9 [7–10]	0 (-1, 0)	0.163
Satisfaction, 0–10	8 [5–10]	7 [5–9]	0 (0, 1)	0.382
Discomfort, 0–10	6 [3–8]	7 [3–9]	0 (-2, 1)	0.574
NOSE score, 0–20	15 [9–18]	15.5 [11–19]	-1 (-3, 1)	0.342

Values are the median [IQR]

Moderate pain and severe pain (items of the QoR15-K) were evaluated using an 11-point numeric rating scale, 0 to 10: 0 = “none of the time” to 10 = “all of the time.”

NOSE score indicates the Nasal Obstruction Symptom Evaluation, 0–20

*CI* confidence interval, *IQR* interquartile range, *QoR15-K* Quality of Recovery 15 Korean version

## Discussion

Preoperative education to simulate postoperative nasal packing and improve the patient’s tolerance to fentanyl requirement was not effective in immediate post-surgery. There were also no differences in requests for analgesics other than fentanyl (i.e., opioids other than fentanyl, acetaminophen, NSAID) between the groups.

Our study was based on the recommendation that anesthesiologists need to inform patients preoperatively to breathe through their mouths after nasal surgery [20] and based on the hypothesis that a non-pharmacologic, non-invasive approach would reduce opioid use after surgery [9, 10, 21]. The discrepancy between our findings from a few previous studies which showed the opioid-sparing effect of preoperative education, may be explained by the different types of surgery and nature of pain [9, 10, 22]. In Zhou et al.’ study in colorectal surgery with urethral catheterization, preoperative illustrative education on urethral catheterization may have helped patients not to be anxious about discomfort and urge to urinate, and better tolerate them [9]. However, in our patients, fear and unbearable sensation originating from nasal fullness accompanied by headache and a difficult sense of breathing may have caused poor adherence to the preoperative education contents [23]. Other studies in trauma and cardiac surgery showed that preoperative education did not reduce opioid consumption in patients with major wounds [24, 25].

Another possible reason for the contrasting results is the difference in education methodology. The education timing and duration may have affected the effect of education. In the study of Zhou et al. in colorectal surgery, education was carried out repeatedly over several days with illustration [9]. In our study, we used simulated training instead of illustration, but education sessions were taken on the night before surgery and the day of surgery immediately before entering the operating room.

A recent meta-analysis showed that psychological preparation may be helpful for postoperative pain, behavioral recovery, and negative affect, although with a low quality of evidence because of heterogeneity of analyzed surgery [22]. The psychological preparation includes providing information on the procedure, sensation (what the experience will feel like and what sensation they may experience), behavioral instruction (what patients need to do), cognitive intervention (change in thinking), relaxation technique (reduce tension and anxiety, relaxation of muscle, breathing technique, guided imagery), hypnosis and emotion-focused intervention (managing feelings) [22]. Among these, our training with nasal clips provided procedural and sensory information. While the similar nasal-obstruction training in nasal surgery effectively reduced emergence agitation in few previous studies, in which cases pain and discomfort are also risk factors for emergence agitation [11, 26], the education intended for opioid reduction after nasal surgery may warrant a different approach.

The education targeted to change the patients’ insight into pain and analgesia request, and clinicians’ awareness for multimodal analgesia may be helpful. In Parsa et al.’s study, they focused on reinforcing the patients’ cognition on the role of endogenous beta-endorphin in analgesia after breast augmentation surgery, and how opioids can hinder its action and reduce mood of well-being [10]. In their study, during the postoperative 7 days, patients were encouraged to take acetaminophen if pain intensity was mild to moderate, and opioids if pain was moderate to severe. In their result, the consumption of acetaminophen was greater than acetaminophen-opioid combined drugs in educated group compared to not-educated group, with better self-reported pain scores and sense of well-being. In our study, we did not differentiate the choice of analgesics (opioids vs. non-opioids) according to pain intensity and did not specifically instruct nor guide the patients on the request of analgesics at the PACU. In future study, incorporating different analgesic choices according to pain intensity, in adjunct to simulated nasal-obstruction training in nasal surgery may reduce opioid consumption.

The strength of our study is that we tried to investigate preoperative non-pharmacologic, simulated training to affect postoperative patient care. Preoperative psychological preparation and simulated training is not harmful, and may potentially be helpful for postoperative outcome [22]. Although we did not show the opioid-reducing effect of simulated training, continuous efforts to develop effective non-pharmacologic, educational approaches and patient-physician interaction may be of worth. Patients undergoing nasal surgery often accompany obstructive sleep apnea (OSA), and they are more susceptible to apnea or hypoxemia from the administration of opioids or sedative drugs. Nasal packing can further obstruct nasal passages and put these patients in particular need of careful postoperative respiratory monitoring. Therefore, along with multimodal analgesia, further research on preoperative education would need to developed and continued.

This study has several limitations. First, this study was conducted at a single center with a Korean population, which may limit the generalizability to a broader population. Pain perception and discomfort are subjective experiences influenced by various factors, including culture, individual experiences, and personality traits. These differences encompass coping mechanisms towards pain, individual differences in pain tolerance, and diverse responses to the education intervention. Second, because the simulation and education were conducted on the evening before the surgery, patients might not have had enough time to fully conceptualize it. When conducting preoperative education, it would be beneficial to consider personalized education methods, such as the use of cartoons or explanatory texts, and allow enough time and repeated exposure to educational content, instead of intensive education immediately before surgery. Third, the preoperative level of anxiety was not evaluated in our patients, which could have affected the immediate postoperative need for opioids, because the prevalence of depression and anxiety is relatively high in patients with chronic sinus symptoms [27]. Incorporation of relaxation techniques or emotion-focused interventions into future education efforts may be beneficial [28].

In conclusion, our intensive preoperative mouth breathing training and education did not improve patient comfort or reduce opioid requirements. However, further studies with a modified explanation approach might be warranted.

### Electronic supplementary material

Below is the link to the electronic supplementary material.

Supplementary Material 1

## Data Availability

The datasets used and/or analyzed during the current study are available from the corresponding author on reasonable request.
