# Supplementary material for: Effect of preoperative patient education and simulated mouth breathing training on opioid requirements in the post-anesthesia care unit after nasal surgery: a randomized controlled study
Source: BMC Anesthesiol. 2023 Oct 20;23:348. doi: 10.1186/s12871-023-02310-x (PMC10588134; doi:10.1186/s12871-023-02310-x)
Supplement: Supplementary file 1 — Supplementary Material 1 [file 12871_2023_2310_MOESM1_ESM.docx]

**Table S1.** Univariable and multivariable logistic regression model for opioid.

|  | | **Total number** | **Opioid,**  ***n* (%)** | **Univariable model** | | | **Multivariable model** | | |
| --- | --- | --- | --- | --- | --- | --- | --- | --- | --- |
|  |  |  |  | **OR** | **95% CI** | ***p* value** | **Adjusted OR** | **95% CI** | ***p* value** |
| Group | Training | 51 | 26 (51.0) | 1.59 | (0.73, 3.45) | 0.245 | 1.80 | (0.76, 4.27) | 0.185 |
| Sex | Female | 36 | 16(44.4) | 0.95 | (0.42, 2.15) | 0.911 |  |  |  |
| Ever smoker, yes | | 43 | 20 (46.5) | 1.10 | (0.50, 2.40) | 0.821 |  |  |  |
| Estimated blood loss, mL | | 104 | 47 (45.2) | 1.00 | (1.00, 1.00) | 0.111 | 1.00 | (1.00, 1.00) | 0.020 |
| Nasal packing site | Unilateral | 22 | 7 (31.8) | reference |  |  |  |  |  |
|  | Bilateral | 82 | 40 (48.8) | 2.04 | (0.75, 5.53) | 0.156 |  |  |  |
| Packing materials | Nasopore | 83 | 33 (39.8) | reference |  |  | reference |  |  |
|  | Beschitin | 9 | 8 (88.9) | 12.12 | (1.45, 101.47) | 0.021 | 19.49 | (2.22, 171.47) | 0.007 |
|  | Both | 8 | 4 (50.0) | 1.52 | (0.35, 6.49) | 0.575 | 2.03 | (0.45, 9.16) | 0.359 |
|  | Others | 4 | 2 (50.0) | 1.52 | (0.20, 11.29) | 0.685 | 3.61 | (0.38, 25.39) | 0.291 |

Values are number of patients (%).

CI: confidence interval; OR: Odds Ratio.
